# Diet, vegetarian food and prostate carcinoma among men in Taiwan

**DOI:** 10.1038/sj.bjc.6602809

**Published:** 2005-10-04

**Authors:** Y C Chen, C I Chiang, R S Lin, Y S Pu, M K Lai, F-C Sung

**Affiliations:** 1 Chia Nan University of Pharmacy and Science, 60 Erh-Jen Road, Jen Te, Tainan, Taiwan 717, Taiwan; 2 Institutes of Environmental Health, National Taiwan University College of Public Health, Taipei, Taiwan 100, Taiwan; 3 Preventive Medicine, National Taiwan University College of Public Health, Taipei, Taiwan 100, Taiwan; 4 Department of Urology, National Taiwan University College of Medicine, 1 Jen-Ai Road Section 1, Taipei, Taiwan 100, Taiwan; 5 Institute of Environmental Health, China Medical University College of Public Health, 91 Hsueh-Shih Road, Taichung, Taiwan 404, Taiwan

**Keywords:** body mass, case–control study, local vegetarian food, prostate neoplasm, Taiwan

## Abstract

In a case–control study in a veterans hospital in Taiwan, we compared 237 histology-confirmed prostate carcinoma cases with 481 controls, frequency matched by age, for their consumption of vegetarian food, namely soybean products, rice, wheat protein and other vegetables. The multivariable logistic regression analysis showed a significant association with such food (odds ratio (OR)=0.67, 95% confidence interval (CI)=0.47, 0.94). This beneficial effect presented for men with body mass index (BMI) ⩽25 kg m^−2^ (OR=0.50, 95% CI=0.32, 0.76) but not for men with greater BMI. The OR of prostate carcinoma for men with BMI ⩽25 kg m^−2^ was 1.74 (95% CI=1.21, 2.51), compared with men with higher BMI (>25 kg m^−2^). Other significant risk factors associated with the disease included higher income (OR=2.40, 95% CI=1.07, 5.42), physical activity (OR=1.75, 95% CI=1.08, 2.83), being married (OR=2.49, 95% CI=1.40, 4.43) and coffee consumption (OR=1.88, 95% CI=1.07, 3.30). Stratified analysis also showed that the consumption of fish/shellfish had an adverse association for men with higher BMI. This study suggests that the intake of the low fat local vegetarian food has a protective effect against prostate carcinoma for thin men in this study population.

Diet has been associated with cancers of the prostate, colorectum, breast and other sites (Doll and Peto, 1981). Although potential dietary risk factors for high-fat and low-fibre foods have shown associations with prostate carcinoma in Western countries (Howell, 1974; Giovannucci *et al*, 1995; Whittemore *et al*, 1995; Parkin *et al*, 1999; Bostwick *et al*, 2004), their role remains unclear for Asian men. Investigation of the disease in populations with lower incidence may provide further insight into its aetiology with relation to diet, particularly as the food consumed in Taiwan is different from that in Western countries.

Asian migrants to the US have shown an appreciable rise in prostate carcinoma incidence, perhaps due to their increasingly westernised lifestyle in recent decades. The incidence of prostate carcinoma in Taiwan increased from 2.3 per 100 000 in 1985 to 12.8 per 100 000 in 1999 *vs* 118.2 per 100 000 in men in the US in 1992–1996 (Ries *et al*, 1999; Department of Health, 2002). This increase is generally attributed to the westernised lifestyle and prostate-specific antigen (PSA) testing in urology clinics in Taiwan. However, despite the increasing incidence in Taiwan, it remains relatively rare as compared with Western countries.

A prospective Hawaiian study (Severson *et al*, 1989) found that a decreased risk of the disease was associated with a high consumption of rice or tofu, which are common low-fat items in Chinese meals, including local vegetarian food in Taiwan, with various products derived from soybean and wheat protein, together with nuts, peas, vegetables and pickles. This study investigated the association of prostate carcinoma with the consumption of such food, which has not previously been evaluated (Sung *et al*, 1999).

## MATERIALS AND METHODS

### Subjects

The cases studied were men, aged 50 years or older, with histology-confirmed prostate adenocarcinoma, and newly identified in the Division of Urology, Department of Surgery at a veterans medical centre in Taipei, Taiwan between August 1996 and July 1998. A total of 259 cases were identified, of whom 22 patients refused to participate or did not complete the interview.

Eligible controls were patients admitted to the same hospital for causes other than malignant diseases. Controls with benign prostate hyperplasia, cardiovascular disease or hormonal disorders were also excluded from this study. Patients with inguinal hernia and renal stones were not excluded. For each case, two hospital controls were selected by frequency match, and were aged 50 years or older. Among the 518 controls, 481 completed the interview, including patients from the Division of Orthopedics and/or Emergency Services (40.5%), Department of Ophthalmology (38.5%) and other departments (21.0%).

### Data collection

With the consent of the subjects, interviews were conducted in person by trained interviewer using standard questionnaires while the participants were in hospital. Adapted from a previous pilot study (Sung *et al*, 1999), the questionnaires included sociodemographic characteristics, history of occupational chemical exposure, tobacco and alcohol use, diet, physical activity, height and weight history, medical history and family history of prostate cancer. Dietary intake history was determined mainly using a semiquantitative food frequency questionnaire. The questionnaire asked for the most frequently consumed foods: pork, beef, fish and shellfish, eggs, vegetables, fruits, carrots, dairy products and entrails, etc. Subjects were asked to recall the consumption of each food item in the past 10 years and determine, compared to other male acquaintances, whether they consumed a greater amount, a moderate amount, less or none at all. The questionnaire also contained some beverages including milk, coffee, soybean milk, rice milk and tea, etc. Daily frequencies and years of drinking and coffee and tea consumptions were measured.

One question specifically asked was about the subjects' consumption patterns of the local vegetarian food, named ‘Zhai’ or ‘Sue’ food in Chinese, abstaining from meat for all meals or for every first day and 15th day each month. We also asked them to determine whether they consumed this type of food in greater, moderate, less amount or none at all. The local vegetarian food in Taiwan is a commercially available unique style of food consisting mainly of products made of soybean and protein of wheat flour. Peanuts, beans, peas, pickles and vegetables stir-fried with vegetable oil are other frequently consumed items in this kind of meals. Plain cooked rice is usually the main staple in a vegetarian meal. Rice soup is often served for breakfast. Buddhist families (not necessarily priests, monks or nuns) are most likely to consume this type of food. Other lay people also consume this local vegetarian food, particularly for breakfast, but may include milk and/or eggs, such as preserved eggs (thousand-year eggs in lay language) or boiled salted eggs.

The major items of physical activities in the questionnaire included leisure-time exercises, work or leisure-time hobbies such as gardening, yard work, carpentry and other domestic chores requiring physical exertion. These were scored and then categorised as sedentary, low, moderate or high.

### Data analysis

The data analyses began with distributions of responses and risk factors to estimate differences in cases and controls. For the comparison of age, subjects were grouped into two groups (<70 *vs* ⩾70 years) based on the age of cases and controls at diagnosis. In this study, participants were mainly veterans (83%). Since the tumorigenesis may have association with the body weight, body mass indexes (BMI) values taken 1 year prior to the interview about the disease and at ages 40–45 years were used in the data analysis and also grouped into two levels (>25 *vs* ⩽25 kg m^−2^) based on the normal BMI range for Taiwanese males. Physical activity was considered as the combination of work and exercises and was classified into two levels (low/moderate *vs* high).

Univariate analyses were used to identify the potential covariates between the folk style vegetarian food and the risk of prostate carcinoma. The strength of the association between prostate carcinoma and potential risk factors was measured, using logistic regression models, in terms of odds ratios (OR) and 95% confidence interval (CI). Significant variables identified from the univariate analyses were included as covariates in multivariable logistic regression models. With age and BMI being included, the downward stepwise modelling was used to determine the best predictors of risk for prostate carcinoma. The least significant variables were deleted one at a time. The final model was derived based on significance testing. Data were analysed using SAS for Windows Version 8.0 (SAS Institute, Inc., Cary, NC, USA).

## RESULTS

More than half of the subjects were aged 70 years or older and approximately 64 percent of participants were retired. Compared with controls, cases had higher education, higher rate of marriage, more children, higher proportion of religious affiliation and a higher family income (Table 1). Cases also had higher physical activity levels than controls. However, cases had a lower BMI than controls. Fewer cases than controls were current smokers. Only a small portion of subjects drank coffee, but the use was significantly higher in cases than in controls (*P*=0.02).

There were no significant differences between cases and controls in the consumption of most dietary beverage variables (Table 2). Cases consumed Taiwanese folk style vegetarian food more often than controls did (65.5 *vs* 55.3%; OR=0.66, 95%, CI=0.48, 0.91). Although a larger percentage of cases drank tea and milk than controls, these differences were not significant either.

Risk factors for prostate carcinoma that remained significant following multivariate conditional logistic regression without interaction terms were higher income, pursuing physical activities, being married, drinking coffee and partaking of folk style vegetarian food (Table 3). Men with high BMI were at an elevated risk of the disease (OR=1.74, CI=1.21, 2.51). The consumption of the folk style vegetarian food showed a significant protective effect (OR=0.67, 95% CI=0.47, 0.94). This protective effect was modified by BMI, significant for men with lower BMI (OR=0.50, 95% CI=0.32, 0.76), but not for men with higher BMI (OR=1.25, 95% CI=0.66, 2.37).

## DISCUSSION

The present case–control study found prostate carcinoma to be negatively associated with the consumption of Taiwanese folk style vegetarian food and BMI, but positively associated with income, being married, coffee consumption and physical activities. Different from other studies (Whittemore *et al*, 1995), this study found no significant increase in the meat intake among cases.

Previous studies on the relationship between physical activities and prostate carcinoma have yielded conflicting results (Lee *et al*, 1992; Ilic *et al*, 1996). Exercise among the elderly in Taiwan is usually a leisure-time activity among the affluent. Physical activities may also be related to a lower BMI. One cohort study and one case–control study have supported a relationship between heavy weight, high BMI and increased risk of prostate carcinoma (Chyou *et al*, 1994; Gronberg *et al*, 1996). Some case–control studies suggested an absence of this association (Whittemore *et al*, 1995; Meyer *et al*, 1997). We found that, with BMI and the folk vegetarian food as interaction terms in the model, body weight appears to have a strong effect, modifying the protective effect of the folk food. To our knowledge, this is an interesting issue that has not been reported. It is not clear why the beneficial effect of Taiwanese folk vegetarian food on prostate carcinoma is limited to thin men.

Giovannucci *et al* (1993) strongly suggested that dietary fat, in particular, the fatty acid in red meat, increases the risk of prostate tumorigenesis two-fold. Several other studies also have demonstrated a similar fat intake association (Gann *et al*, 1994; Vlajinac *et al*, 1997).

A comprehensive case–control study found a strong trend of risk with fat intake in all ethnic groups, including for Asian-American men (Whittemore *et al*, 1995). It suggests an increase in prostate carcinoma risk as men migrate from a low dietary-fat to high dietary-fat culture. The incidence of prostate carcinoma for Chinese men in Taiwan (Department of Health, 2002) is far lower than that for Chinese Americans (Ross *et al*, 1981). This is probably due to a higher intake of dietary fat from red meat by Chinese Americans than by men in Taiwan. This may also be partly due to more PSA tests in the US than in Taiwan. There was a long period of economic hardship in Taiwan prior to the industrial development in the 1980s. Increasing consumption of meat began in the 1980s and 1990s. The per capita daily intake of energy from fat in Taiwan increased from 22% in 1970 to 28% in 1980 and to 42% in 1998 (Directorate-General of Budget, 1999). Economic change and longer life expectancy might have an impact on prostate tumorigenesis for men in Taiwan and may explain partly the tripled incidence increase in the last decade.

It is also possible that the greater use of PSA tests in the 1990s for patients with urological complaints may partly explain the increase of the disease in Taiwan. However, no community-based PSA screening has been conducted yet in Taiwan.

Earlier studies have reported an increased risk of prostate carcinoma for men with more children or men who are married (Armenian *et al*, 1975; Greenwald *et al*, 1994). Nevertheless, Catholic priests experience no decreased mortality from prostate carcinoma (Ross *et al*, 1981). Our results showed that men who married or fathering children were at increased risk of developing prostate carcinoma. Most veterans in Taiwan in general get married at later ages and have fewer children or are not married at all because of military service. The higher marital rate and higher rate of being a father in the cases may have been due to the effects of economic prosperity that made having a family easier than for the controls. Some cases married at an earlier age than controls.

Fat intake has been generally thought to increase serum sex hormone levels that may have an association with prostate carcinoma (Parker *et al*, 1998). The main fat intake in Taiwan is from pork instead of beef. The intake of pork and other types of meat was not significantly different between cases and controls in the present study, neither was the difference in ordinary vegetable consumption.

Nevertheless, the folk vegetarian food provides a protective effect against prostate carcinoma. The tofu and other soybean products, wheat protein, beans, nuts, pickles and plain rice, etc. in the folk vegetarian meals are normally prepared as a ‘light’ type of diet characterised by less amounts of fat, and no meat and animal fat. About 55% of cases and 65% of controls reported being frequent consumers of folk vegetarian meals. Chinese Americans may not have this type of vegetarian food as much as Chinese in Taiwan do. This study showed that men who consumed Taiwanese folk style vegetarian food had a 50% reduced risk of prostate carcinoma for thin men. Further analysis showed that the consumption of egg and milk together with this type of folk food remains the beneficial effect (data not shown).

The protective effect of the vegetarian meals against the development of prostate carcinoma in this study is consistent with findings in some other studies on the consumption of selected vegetables. Those studies suggest that increased consumption of green leafy vegetables, soybean products, lentils and peas, and tomatoes are associated with decreased prostate carcinoma risk (Mills *et al*, 1989; Key *et al*, 1997). These foods are also components served in the Taiwanese folk vegetarian meals. A prospective study of Japanese-Americans in Hawaii suggested that consumption of rice and tofu decreased the risk of prostate carcinoma (Severson *et al*, 1989). This type of Japanese-American meal is also light, somewhat similar to the Taiwanese folk vegetarian meals. Adlercreutz (1995) has indicated that lignins and the isoflavonoids in soybeans, whole-grain products, various seeds and other similar food components may have an influence on sex hormone metabolism and biological activity. These food items also have an influence on intracellular enzymes, protein synthesis, growth factor action, malignant cell proliferation and angiogenesis, making them protective against carcinoma. It has also been found that vegetarians with high dietary fibre intake excrete large amounts of sex steroids in the faeces, thereby lowering the levels of sex hormones in the plasma (Howie and Schultz, 1985). One of the sex steroids, testosterone, is essential for the normal growth of prostate epithelium, and early stage prostate carcinoma has been shown to be endocrine dependent (Pienta and Esper, 1993).

Some limitations to the present study should be considered. First, recall bias is a drawback in case–control studies. The use of hospital-based controls could decrease differences in health consciousness between cases and controls although using hospital-based controls may also minimise the effect of risk factors. Second, the use of semiquantitative food frequency to quantify diet may not reveal the precise effect of risk factors such as meat and fat consumption. However, its use is very convenient for the elderly with little or no education who must recall the contents of their diet. On the other hand, the folk vegetarian food termed as ‘Zhai’ or ‘Sue’ food in Taiwan is easily recalled and counted by lay people regardless of educational levels. Third, due to the use of a binary analysis regarding all risk factors, we might have missed potentially important information such as a dose response. The use of semiquantified data restricts our ability to stratify data into multiple levels. It is also likely that subjects may have under-reported their exposure status, but such bias would likely be similar in both cases and controls.

This case–control study showed that prostate carcinoma cases were more likely to occur in educated individuals who engaged in more physical activities and had a lower body mass index. The seemingly protective effect related to the increased intake of folk vegetarian food with very low fat content is particularly significant for thin men.

## Figures and Tables

**Table 1:** Sociodemographic characteristics and lifestyle of prostate carcinoma cases and controls in Taiwan, 1996–1998

**Characteristic**	**Cases *N*=237** ***n* (%)**	**Controls *N*=481** ***n* (%)**	***P*-value^a^**
*Age, years*			
Mean±s.d.	72.2±6.1	71.1±5.8	0.03
<70	95 (40.1)	220 (45.7)	0.15
⩾70	142 (59.9)	261 (54.6)	
			
*Veterans*			
No	42 (17.8)	79 (16.5)	0.66
Yes	194 (82.2)	400 (83.5)	
			
*Education, years*			
⩽6	76 (32.1)	227 (47.2)	0.001
7–12	87 (36.7)	150 (31.2)	
>12	70 (29.5)	97 (20.2)	
Unknown	4 (1.7)	7 (1.4)	
			
*Married*			
No	17 (7.2)	78 (16.2)	0.001
Ever married	220 (92.8)	401 (83.4)	
Unknown	0 (0.0)	2 (0.4)	
			
*Having children*			
Yes	207 (87.3)	380 (79.0)	0.003
No	27 (11.4)	99 (20.6)	
Unknown	3 (1.3)	2 (0.4)	
			
*Religious*			
Buddhism/Taoism	88 (37.1)	152 (31.6)	0.11
Others/none	145 (61.2)	326 (67.8)	
Unknown	4 (1.7)	3 (0.6)	
			
*Occupation*			
Retired	151 (63.7)	306 (63.6)	0.3
Government employee	19 (8.0)	51 (10.6)	
Business	50 (21.1)	97 (20.2)	
Others	16 (6.7)	19 (3.9)	
Unknown	1 (0.5)	8 (1.7)	
			
*Family income*			
Low/moderate	210 (88.6)	420 (87.3)	0.005
Higher	16 (6.8)	11 (2.3)	
Unknown	11 (4.6)	50 (10.4)	
			
*BMI, at age 40–45 years*			
⩽25 kg m^2^	173 (73.0)	323 (67.2)	0.11
>25 kg m^2^	64 (27.0)	158 (32.8)	
			
*BMI, 1 year before interview*			
⩽25	165 (69.6)	285 (59.3)	0.007
>25	72 (30.4)	196 (40.7)	
			
*Physical activity*			
Low/moderate	196 (82.7)	425 (88.4)	0.02
Higher	389 (16.0)	49 (10.2)	
Unknown	3 (1.3)	7 (1.4)	
			
*Smoking*			
Yes	64 (27.0)	164 (34.1)	0.05
Ever smoking	93 (39.2)	147 (30.6)	
No	80 (33.8)	170 (35.3)	
			
*Alcohol*			
Yes	61 (25.7)	142 (29.5)	0.32
No	174 (73.5)	339 (70.5)	
Unknown	2 (0.8)	0 (0.0)	
			
*Coffee*			
Yes	31 (13.1)	36 (7.5)	0.02
No	206 (86.9)	443 (92.1)	
Unknown	0 (0.0)	2 (0.4)	
*Tea*			
Yes	113 (47.7)	208 (43.2)	0.26
No	124 (52.3)	273 (56.8)	
			
*Any chemical exposure*			
Yes	74 (31.2)	161 (33.5)	0.57
No	162 (68.4)	320 (66.5)	
Unknown	1 (0.4)	0 (0.0)	

a Chi-square tests except using *t*-test for the comparison of means.

s.d.=standard deviation.

BMI=body mass index, kg m^−2^.

**Table 2:** Odds ratios (95% confidence intervals) for selected dietary consumption variables for prostate carcinoma among men in Taiwan, 1996–1998

	**Cases *N*=237** ***n* (%)**	**Controls *N*=481** ***n* (%)**	**Crude OR (95% CI)**	**Age-adjusted OR (95% CI)**
*Pork*				
⩾Moderate	191 (80.6)	373 (77.6)	1.18 (0.8,1.74)	1.24 (0.84,1.84)
Less, none	46 (19.4)	106 (22.0)	1.0	1.0
Unknown	0 (0.0)	2 (0.4)		
				
*Beef*				
⩾Moderate	154 (65.0)	308 (64.1)	1.04 (0.75,1.43)	1.05 (0.76,1.46)
Less, none	83 (35.0)	172 (35.7)	1.0	1.0
Unknown	0 (0.0)	1 (0.2)		
				
*Egg*				
⩾Moderate	178 (75.1)	360 (74.9)	1.00 (0.7,1.43)	1.01 (0.70,1.44)
Less, none	59 (24.9)	119 (24.7)	1.0	1.0
Unknown	0 (0.0)	2 (0.4)		
				
*Fish/shellfish*				
⩾Moderate	161 (67.9)	317 (65.9)	1.09 (0.78,1.52)	1.12 (0.80,1.56)
Less, none	76 (32.1)	163 (33.9)	1.0	1.0
Unknown	0 (0.0)	1 (0.2)		
				
*Vegetables/fruits*				
⩾Moderate	212 (89.5)	428 (89.0)	1.03 (0.62,1.71)	1.03 (0.62,1.71)
Less/none	25 (10.5)	52 (10.8)	1.0	1.0
Unknown	0 (0.0)	1 (0.2)		
				
*Folk Sue food*				
⩾Moderate	131 (55.3)	315 (65.5)	0.66 (0.48,0.91)	0.66 (0.48,0.91)
Less, none	100 (43.0)	159 (33.0)	1.0	1.0
Unknown	6 (2.5)	7 (1.5)		
				
*Dairy products*				
⩾Moderate	104 (43.9)	187 (38.9)	1.23 (0.89,1.68)	1.20 (0.87,1.65)
Less, none	132 (55.7)	291 (60.5)	1.0	1.0
Unknown	1 (0.4)	3 (0.6)		

OR=odds ratio; CI=confidence interval; Folk Sue food=folk-style vegetarian food.

**Table 3:** Odds ratios (95% confidence intervals) for variables associated with prostate carcinoma among men in Taiwan estimated from multivariate logistic regression models

	**Total** **OR (95% CI)**	**BMI>25 kg m^−2^** **OR (95% CI)**	**BMI⩽25 kg m^−2^** **OR (95% CI)**
*Age, years*			
⩾70	1.42 (1.00,2.01)	1.23 (0.67,2.25)	1.45 (0.94,2.24)
<70	1.0	1.0	1.0
			
*Income*			
High	2.40 (1.07,5.42)	2.61 (0.67,10.2)	2.62 (0.91,7.50)
⩽Moderate	1.0	1.0	1.0
			
*Physical activity*			
High	1.75 (1.08,2.83)	1.63 (0.68,3.90)	1.84 (1.01,3.34)
⩽Moderate	1.0	1.0	1.0
			
*BMI (kg m*^−*2*^)			
⩽25	1.74 (1.21,2.51)	—	—
>25	1.0		
			
*Married*			
Yes	2.49 (1.40,4.43)	2.29 (0.64,8.23)	2.74 (1.42,5.30)
No	1.0	1.0	1.0
			
*Coffee*			
Yes	1.88 (1.07,3.30)	2.34 (0.91,6.04)	1.91 (0.93,3.95)
No	1.0	1.0	1.0
			
*Folk vegetarian food*			
⩾Moderate	0.67 (0.47,0.94)	1.25 (0.66,2.37)	0.50 (0.32,0.76)
Less/none	1.0	1.0	1.0
			
*Fish, shellfish*			
⩾Moderate	1.15 (0.79,1.66)	2.45 (1.14,5.24)	0.87 (0.56,1.36)
Less/none	1.0	1.0	1.0

OR=odds ratio; CI=confidence interval; BMI=body mass index a year prior to diagnosis.
